# Microfluidic Femtosecond Laser-Induced Nucleation of Supersaturated Aqueous Sodium Chlorate Solutions

**DOI:** 10.1021/acsomega.4c11633

**Published:** 2025-06-23

**Authors:** Liye Yang, Yoichiroh Hosokawa, Ming Li, Yuka Tsuri, Shaokoon Cheng, Yaxiaer Yalikun

**Affiliations:** 1 Division of Materials Science, Graduate School of Science and Technology, 12708 Nara Institute of Science and Technology, Ikoma 630-0192, Japan; 2 School of Engineering, Macquarie University, Sydney, NSW 2109, Australia; 3 School of Mechanical and Manufacturing Engineering, 7800 University of New South Wales, Sydney, NSW 2052, Australia

## Abstract

Femtosecond laser-induced crystallization has gained significant attention due to its precise control over crystal formation in recent years; however, challenges remain in improving the efficiency and consistency of this process. Another emerging approach for crystallization is the use of microfluidic systems, where strategies such as solvent exchange, temperature modulation, and evaporation have been explored; however, achieving consistent and localized nucleation remains an active area of investigation. In this study, the probability of crystal enhancement through the integration of femtosecond laser-induced crystallization with microfluidic chip technology was investigated. A microfluidic device with a channel width of 3 mm, capable of continuous femtosecond laser pulse irradiation, was designed to control crystal number and size growth under a controlled flow rate. Sequentially, tunable crystal generation in the microfluidic chip was realized by irradiating the channel with femtosecond laser pulse supersaturated sodium chlorate (NaClO_3_) solution as low threshold as σ = 0.002. The size and number of crystals could be tuned by the supersaturation and flow rate of the sample solution, the laser pulse energy, and the number of laser pulses.

## Graphical abstract

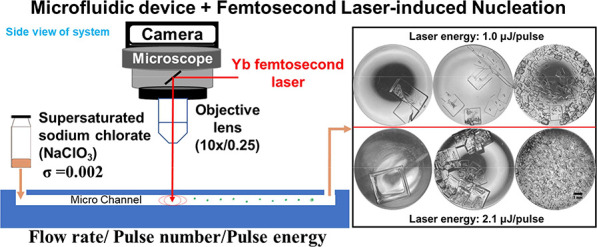

## 1 Introduction

Crystallization from solution is widely recognized in both scientific and commercial applications as an effective technique for concentrating and purifying chemicals^1,2^ To induce crystallization, solution conditions such as concentration and temperature need to be carefully controlled; however, by themselves, it is generally difficult to precisely control when and where nucleation occur. To achieve spatiotemporal control of crystallization, various laser-induced crystallization techniques have gained considerable attention. Since Garetz et al. first reported the laser-induced crystallization of urea in 1996, numerous photophysical and photochemical methods for crystallization have been proposed, utilizing a wide range of laser sources.^3–10^

One of the most significant breakthroughs in laser processing was femtosecond laser-induced crystallization. Unlike nanosecond or picosecond laser excitation, where enhanced molecular and lattice vibrations are effectively transferred to the surrounding environment, resulting in significant heat transfer from the irradiated area, femtosecond laser ablation operates through a different mechanism. Femtosecond laser ablation is governed by a photomechanical mechanism driven by transient pressure rather than heat, minimizing the heating effect. Femtosecond laser pulses focused into a solution form micrometer-sized cavitation bubbles that expand and contract in microseconds, which has been proposed to be the trigger for crystallization.^7^ Cavitation bubbles generated in low-supersaturation solutions are presumed to increase the local concentration around them, inducing crystal nucleation. This technique is particularly beneficial for high-quality protein crystallization, as femtosecond irradiation helps prevent thermal decomposition and denaturation.^11,12^ Furthermore, our previous work succeeded in controlling crystal polymorphs of organic molecules.^13^ Crystallization by femtosecond laser irradiation is expected to be applied to a variety of materials. However, the significant and obvious challenges of this technique are the quantitatively predicting and finite size.^14^ The limitations of this technique include challenges in quantitatively predicting the crystallization outcomes, such as nucleation probability and the crystal growth rate, as well as the constraints imposed by the finite volume of traditional crystallization systems. In addition, the crystallization process resembles a batch setup, where only a small portion of the solution is exposed to laser light at a time rather than a continuous system where the entire solution is processed simultaneously.

When it comes to control efficiency, microfluidic technology, as an emerging technique, is receiving increasing attention. Its applications in crystallization are also becoming more widespread. Microfluidics has revolutionized biological and chemical research by introducing unique experimental techniques with in situ analytics.^15,16^ Compared to traditional batch methods, microfluidic systems offer superior heat and mass transfer, isolation from potential contaminants, and reduced material usage. These advantages enable the generation of significantly higher supersaturations and allow for precise control over crystal nucleation.^17,18^ The application of microfluidics in crystallization has been extensively explored in the synthesis of pharmaceuticals, nanocrystals, and proteins.^19–22^ Microfluidic crystallization strategies offer unique advantages, including enhanced control over nucleation conditions, reduced reagent consumption, and the potential for integration into continuous manufacturing systems.

Here, we proposed a method to enhance crystallization induced using the combination of microfluidic and femtosecond laser to solve all the problems been mentioned above, which have not been reported.^23–25^ Many studies have reported that microfluidic systems can induce crystallization by adding antisolvents, but the introduction of antisolvents increases the risk of particle contamination and higher solvent residues. In crystallization without additives, methods have been proposed in which physical stimuli, such as ultrasound^14,26^ or laser irradiation, are applied externally to a supersaturated solution.^27^ Laser-induced crystallization is expected to enable precise temporal and spatial control. Previous research has also demonstrated the application of microfluidic systems for laser-induced nucleation using nanosecond lasers.^23,24,28^ These studies established the feasibility of combining laser nucleation with microfluidics. Femtosecond laser-induced crystallization can be advantageous for certain sensitive solutes and solvents, particularly when using shorter pulse durations (∼300 fs), which have been shown to minimize heat accumulation compared with longer pulses. As demonstrated by Tsuri et al.,^29^ shorter pulse durations reduce local temperature increase and enhance nucleation efficiency, thereby lowering the overall laser energy required for crystallization. However, further comparative studies between femtosecond and nanosecond laser-induced crystallization are needed to comprehensively evaluate their effects on solute utilization and thermal stability in various systems.

In this work, we aim to address these challenges by leveraging focused femtosecond lasers, which have low thermal effects due to their high peak intensity, in combination with a microfluidic system. This approach provides greater control over the nucleation dynamics and enhances the reproducibility and scalability of the crystallization process. Sodium chlorate (NaClO_3_) was chosen as the sample in this study due to its crystallization that has been widely studied by easy observation and its suitability as a model compound for current work.

In the present research, we explored microfluidic chip-enhanced femtosecond laser-induced crystallization using supersaturated aqueous sodium chlorate solutions. The desired supersaturation σ = (*C – C*_sat_)*/C*_sat_, where *C* is the solution concentration and *C*_sat_ is the concentration of a saturated solution, ranging from 0.000 to 0.043. Our experimental setup featured a custom-designed microfluidic chip that enabled the continuous flow of supersaturated solutions. Additionally, this design allowed for femtosecond laser irradiation directly within the designated microfluidic channel part, enabling real-time observation of cavitation bubble formation and crystal generation. Furthermore, in this study, the femtosecond laser is focused using a 10× microscope objective with a numerical aperture (NA) of 0.25, resulting in an approximate beam diameter of 21 μm. The use of focused femtosecond laser pulses enhances the nucleation efficiency within the microfluidic system, contributing to reproducible crystallization outcomes under controlled flow conditions. While the laser localizes the initial nucleation event, the downstream processing and postirradiation aging steps mean that spatial and temporal control over nucleation does not directly translate to control over the final crystallization state. Nevertheless, the entire process—including solution injection, filtration, laser-induced nucleation, and collection into glass cylindrical vials—was continuous, resulting in faster and more efficient crystal generation compared to conventional batch methods. Notably, this study represents the first reported use of microfluidic chip-enhanced femtosecond laser-induced crystallization.

## 2 Experimental Section

### 2.1 Supersaturated NaClO_3_ Solution Sample Preparation

NaClO_3_ powder (98.0% purity, FUJIFILM Wako Pure Chemical Corporation) 20.8–21.8 g was dissolved in 20.0 mL of purified water to prepare aqueous NaClO_3_ solutions with concentrations of 50.2–52.2% w/w. The NaClO_3_ was fully dissolved by heating the solution at 70.0 °C for 2 h, followed by filtration using a 0.2 μm filter. After filtration, the solution was cooled to 23.0 °C, resulting in a supersaturated state. Saturation concentration *C*_sat_ is the solubility at 23.0 °C, which is 50.2% w/w.^30^ The supersaturations of solutions were σ = 0.000–0.043. Supersaturated solutions were filtered again into clean glass cylindrical vials (20 mL) with screw caps. All of the vials were cleaned with filtered deionized water before use.

### 2.2 Microfluidic Device Fabrication

The polydimethylsiloxane (PDMS) device was fabricated using a master mold created with a negative photoresist (SU-8 3050, Tokyo Ohka Kogyo, Tokyo, Japan) on a 4 in. silicon wafer using standard soft lithography techniques^31–33^ (Figure 1a and Figure S1). The PDMS (SYLGARD 184, Dow Corning, Midland, MI, USA) was prepared by mixing the base and curing agent in a 10:1 weight ratio. The mixture was poured over the master mold, degassed to remove air bubbles, and cured at 80 °C for 3 h. Once cured, the PDMS microchannel layer was peeled off the mold and cleaned using adhesive tape. Before bonding, both the PDMS layer and a borosilicate glass slide (76 × 26 × 0.8 mm) underwent oxide plasma treatment for 35 s at 65 W using a plasma cleaner (CY-P2L-B, Zhengzhou CY Scientific Instrument Co., Ltd., Zhengzhou, China).

**Figure 1:**
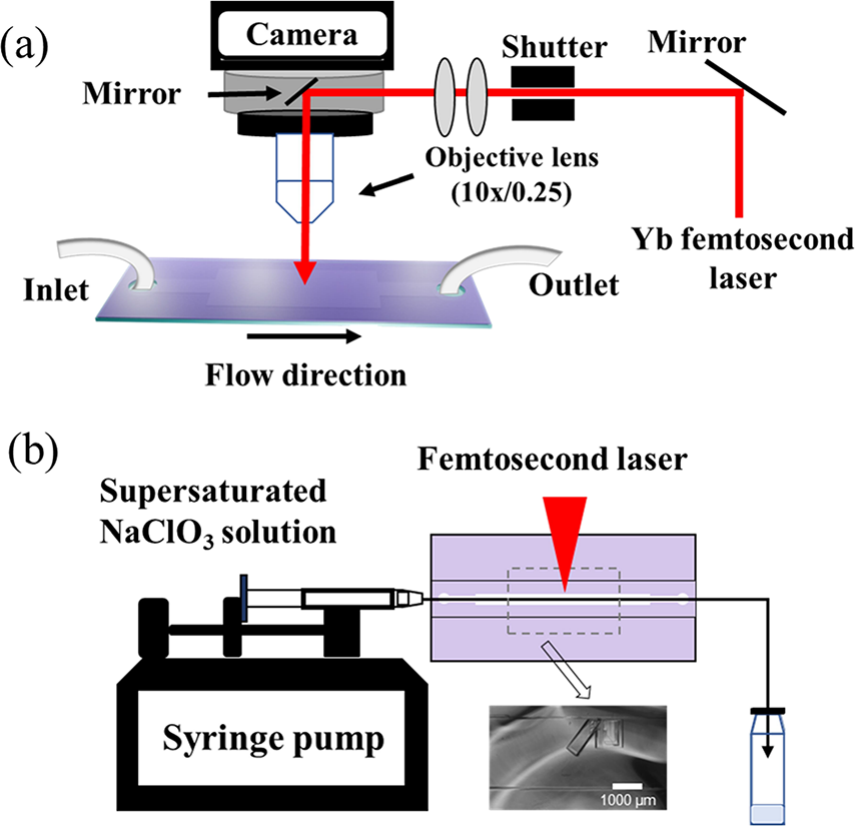
Schematic illustration of experimental apparatus of the microfluidic device-enhanced fs-laser-induced system of the current work. (a) Side view was shown, highlighting the experiment system consisted of Yb femtosecond laser [Spirit one, (Spectra Physics) 1040 nm, 400 fs, 200 kHz, 8 W] and a camera with a 10× objective lens. (b) Top view of the experimental setup was presented; the irradiated part of the channel is 3 mm in width and 0.12 mm in depth. The total channel length is 65 mm. The volume of the microfluidic device is 162 μL. An inset showed a magnified view of the microchannel and crystals alignment under controlled flow conditions.

The resulting microchannel in the detection area measures 3 mm in width and 120 μm in height with 35 mm in length. The microchannel for the inlet and outlet is 1 mm wide, with a 15 mm in length. The sample fluid is pumped through an inlet at different flow rates of 100–2000 μL/min via a syringe pump. The device’s outlet, connected directly to the sealed glass cylindrical vials, helps minimize the risk of solution sample exposure to the air to evaporate the water.

### 2.3 Optical System

A femtosecond laser is a pulsed laser capable of producing ultrashort pulses that emit optical pulses with bursts of laser energy at an extremely fast rate in the domain of femtoseconds (1 fs = 10^–15^ s). The femtosecond laser device used in this setup is from a Spirit One femtosecond laser (Spectra Physics, wavelength: 1040 nm, pulse duration: 400 fs, repetition rate: 200 k to1 MHz, max laser power: 8 W). The femtosecond laser beam was focused using a 10× objective lens (NA = 0.25), resulting in a beam waist diameter of approximately 21 μm. A Collimator, consisting of two lenses placed between the shutter and the mirror, was used to ensure beam collimation before entering the focusing optics. The motorized microscope stage allowed for precise monitoring of the microfluidic flow. Crystals were imaged using a microscope and camera positioned downstream of the irradiation zone, ensuring that the recorded images corresponded to crystallization occurring under flow conditions. The output of these lasers is available to be combined with the microscope and microfluidic systems, as shown in Figure 1.

### 2.4 Experiment Setup and Data Process

As shown in Figure 1b, the supersaturated NaClO_3_ solution could be pumped in the PDMS layer so that the observation zone of the channel functioned as the nucleation zone, where solutions were irradiated by femtosecond laser pulses continuously. Laser irradiation through a PDMS layer would not impact the laser path irradiation effect.^34^ A motorized microscope stage (BIOS-L101S OptoSigma, Tokyo) was used to monitor the solution flow status in the microfluidic chip on an upright microscope (BX53 Olympus, Tokyo). For the NaClO_3_ solution sample flow loading (100–2000 μL/min) in this experiment, a syringe pump was used (Harvard Apparatus, Massachusetts, Holliston) to ensure the accurate and constant speed of the flow rate in this experiment. The experimental data were generated through the following four designs.

#### 2.4.1 Supersaturated Concentration Screening

The experiment was set up as shown in Figure 1b. The supersaturated NaClO_3_ solutions with σ = 0.000–0.0043 were transferred to the glass cylindrical vials via a microfluidic device at 200 μL/min, during which the femtosecond laser was irradiated to the observation zone of the microfluidic channel. The solution in the stock served as the control group. Additionally, samples transferred via the microfluidic chip without laser irradiation and samples irradiated without being transferred via the microfluidic chip were also set as control groups. The control group of samples was irradiated outside the microfluidic device using the same 10× objective lens (NA = 0.25) to ensure consistent laser focusing and energy delivery. This setup replicated the irradiation conditions within the microfluidic device as closely as possible except for the absence of continuous flow. By maintaining identical laser parameters, the experiment isolated the effect of flow dynamics on nucleation and crystal growth. The pulse energy and repetition rate used in this experiment were 1.0 μJ/pulse and 200 kHz, respectively. The crystallization probability was calculated as (*n*_cry_/*n*_total_) × 100. The *n*_total_ is the number of prepared glass vials for each condition. The *n*_cry_ is the number of glass vials in which crystallization was observed under these conditions.^13,35^ The term “Normalized (Number of Crystals)” is used to address the challenges in directly counting crystals, particularly when some are fragmented or too small for accurate enumeration. This provides a standardized approach for reporting crystal counts under different conditions, consistent with practices in related studies^24,28^ In our experiments, after 24 h refers to the postprocessing period after the solution was transferred through the microfluidic device and irradiated with the femtosecond laser. The irradiated samples were then collected in vials and stored at room temperature for 24 h before analyzing the crystallization outcomes.

#### 2.4.2 Investigation of Number of Laser Pulse Dependency

Supersaturated NaClO_3_ solution (σ = 0.010 and 0.015) was transferred via a microfluidic device at a flow rate of 200 μL/min. Pulse energy was set at 1.0 μJ/pulse and the repetition rate at 200 kHz with different total laser pulse numbers at 1.25 × 10^5^, 5.0 × 10^5^, 2.0 × 10^6^, and 8.0 × 10^6^ to irradiate the transferring supersaturated NaClO_3_ solution, respectively, which were realized by adjusting the shutter speed. The shutter speed adjustment was used to control the frequency of laser pulses while ensuring that the total experimental time remained constant at 40 s

#### 2.4.3 Investigation of Pulse Energy Dependency

Supersaturated NaClO_3_ solution (σ = 0.010 and 0.0015) was transferred via a microfluidic device at a flow rate of 200 μL/min. The power of the femtosecond laser was adjusted to study the influence of the crystals. Three different pulse energies (0.4, 1.0, and 2.1 μJ/pulse) were used for comparison.

#### 2.4.4 Investigation of Flow Rate Dependency

supersaturated NaClO_3_ solution (σ = 0.010 and 0.015) was transferred via a microfluidic device at flow rates of 100, 200, 400, 1000, and 2000 μL/min to study the impact on the crystallization by different flow rates. The parameter of femtosecond laser per pulse was set at 1.0 μJ/pulse with frequency at 200 kHz.

## 3 Results and Discussion

### 3.1 Supersaturated Concentration Screening

The experiment results listed in Table 1 and Table S1 were were designed to determine the optimal working conditions for solution sodium chlorate in a microfluidic chip control system. As a control, in the prepared supersaturated conditions, crystallization did not occur without both laser irradiation and flow. Additionally, no crystallization was also observed within 24 h when the laser was irradiated into the bulk solution or when the solution was flowed without laser irradiation. In contrast, crystallization occurred when a solution with supersaturation at σ > 0.005 flowing through a microfluidic device was irradiated with laser pulses. Notably, in supersaturated solutions with σ > 0.010, a crystallization probability nearing 100% was achieved within 24 h.

**Table 1:** Summary of Experimental Results for Different Supersaturation Levels (σ) in the Control, Microfluidic Chip Only, Laser Only, and Microfluidic Chip-Assisted Crystallization Induced by Laser^b^

						microfluidic chip flow and laser irradation, **this method**			
						(200 μL/min, 1.0 μJ/pulse)			
						sample #			
mass of NaClO_3_ in 1 mL H_2_O (g)	concentration (% w/w)	supersaturation σ = (*C – C*_sat_)*/C*_sat_	control (in-stock supersaturated solution)	microfluidic chip flow only (200 μL/min) (no laser irradation)	laser irradation only (1.0 μJ/pulse) (no microfluidic chip flow)	1	2	3	4
1.01	50.2	0.000	clear	-	-	-	-	-	-
1.02	50.5	0.005	clear	-	-	-	-	-	-
1.03	50.7	0.010	clear	-	-	-	-	-	-
1.04	51.0	0.015	clear	-	-	-	-	-	+
1.05	51.2	0.020	clear	-	-	-	+	+	+
1.06	51.5	0.024	clear	-	-	-	-	+	-
1.07	51.7	0.029	clear	-	-	+	-	-	+
1.08	51.9	0.034	clear	-	-	-	-	-	-
1.09	52.2	0.038	clear	-	-	-	-	-	-
1.10	52.4	0.043	clear	-	-	+	+	block	
After 24 h									
1.01	50.2	0.000	clear	-	-	-	-	-	-
1.02	50.5	0.005	clear	-	-	-	-	-	-
1.03	50.7	0.010	clear	-	-	-	+	-	+
1.04	51.0	0.015	clear	-	-	+	+	+	+
1.05	51.2	0.020	clear	-	-	+	+	+	+
1.06	51.5	0.024	clear	-	-	-	+	+	+
1.07	51.7	0.029	clear	-	-	+	+	+	+
1.08	51.9	0.034	clear	-	-	-	+	+	+
1.09	52.2	0.038	clear	-	-	+	+	+	+
1.10	52.4	0.043	clear	-	-	+	+	N/A^a^	

a The microchip channel was blocked. The data from samples 3 and 4 of concentration 52.4% w/w were not applicable.

b “+” represents crystallization, and “-” represents no crystallization. The lower half of the table represents runs where the solution, after passing through the microfluidic device and undergoing laser irradiation, was collected in a vial and allowed to age undisturbed for 24 h at room temperature before crystal size and number were measured.

This result demonstrates for the first time that a femtosecond laser would induce the crystallization by the enhancement effect by a microfluidic chip. Irradiating laser pulses into the bulk solution continuously, the temperature increases at the focal point, which is generally the negative effect for crystallization because of reducing supersaturation.^29^ The reduced nucleation efficiency observed in bulk vials may be attributed to localized heating effects at the laser focal point. While convection in the bulk solution likely mitigates some of the localized heating, limited replenishment at the focal point may still lead to temperature increases that inhibit nucleation. Although we did not measure the temperature directly or test glass vials under lower pulse rates to mitigate cumulative heating effects, these factors warrant further investigation. However, when the solution is flowed, the laser continuously irradiates the fresh solution, effectively mitigating the heating. We propose that, in flowing solutions, the concentration increase due to laser-induced cavitation bubbles effectively induced crystallization even in low-supersaturated solutions that do not crystallize upon laser irradiation alone. However, it was also indicated that crystals would grow too fast to block the microchannel by transferring higher concentration solutions, which was out of control. Thus, the study range was set at σ = 0.005–0.043.

Figure 2a shows the optical image of the crystals observed in glass vials after flowing and laser irradiation. By these images, we measured the number of crystals and crystal size. Figure 2b demonstrates a clear inverse relationship between crystal size and the number of crystals as during σ = 0.000–0.015. At σ= 0.000, no crystals were observed either during the laser irradiation or after 24 h. This is due to the lack of supersaturation in the solution, which prevents the nucleation process from occurring. The 24 h period allowed sufficient time for any potential crystallization to occur, but no such events were observed under these conditions. At σ > 0.005, the crystals were observed, indicating that the number of crystals increases as the supersaturation increases. The crystal size decreases with an increase in the number of crystals. At σ = 0.010 and 0.015, the normalized number and mean crystal size suggest that they might be an optimal balance between nucleation and growth, which might be controlled by changing the certain parameters of the femtosecond laser and microfluidic flow rate. We considered the laser conditions in supersaturated solutions with σ = 0.010 and 0.015, which have a high crystallization probability and allow us to compare the differences in crystal size and number.

**Figure 2:**
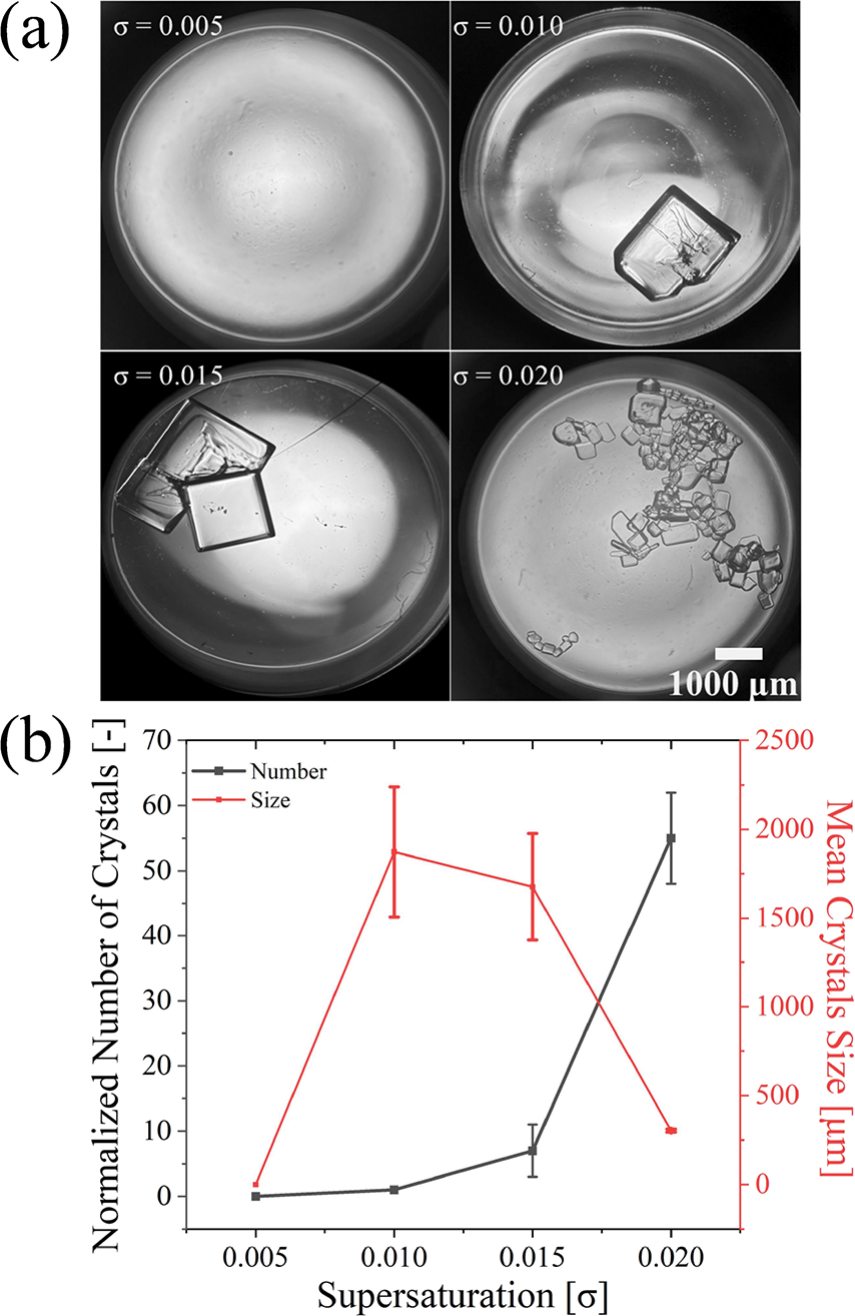
(a) Photos of the crystals in each supersaturated concentration. (b) Plot of crystal number and size distribution vs supersaturation, σ of 0.000, 0.005, 0.010, and 0.015. Threshold supersaturated solution of the generated crystals was σ = 0.005. Error bars indicate standard deviations (*n* = 3).

The combination of laser irradiation and flow was found to significantly enhance crystallization compared to that with flow only or laser irradiation only. This enhancement can be attributed to the synergistic effects of these two factors. Laser irradiation provides localized energy to initiate nucleation, while the flow ensures continuous replenishment of the solute and prevents localized solute depletion. Moreover, the dynamic flow environment minimizes crystal agglomeration by transporting nucleated crystals downstream, leading to the formation of discrete and uniform crystals. These combined effects highlight the advantages of integrating laser-induced nucleation with microfluidic flow systems

### 3.2 Number of Laser Pulse

As shown in Figure 3, with the number of laser pulses increased from 1.25 × 10^5^ to 8.0 × 10^6^, for the solution with σ = 0.010, the average number increased from 3 to 23, and the mean size increased from 1125.0 to 1264.0 μm. For the solution with σ = 0.015, the average number increased from 3 to 23, and the mean size first increased from 729.0 to 1011.0 μm and then further decreased to 823.0 μm. For both concentrations, no crystals were formed when the laser pulse count started at 1.25 × 10^5^. These results indicate that as the number of laser pulses decreases, the number of crystals also decreases, while the crystal size remains relatively stable. This behavior suggests a saturation effect where nucleation reaches a threshold beyond which additional laser pulses no longer proportionally increase nucleation events. In this regime, crystal growth is primarily governed by the availability of solute and mass transport dynamics rather than the absolute number of pulses. Therefore, while a higher number of pulses facilitates more nucleation, the growth competition among crystals is the limiting factor in size determination, leading to a relatively stable crystal size despite variations in the pulse number.

**Figure 3:**
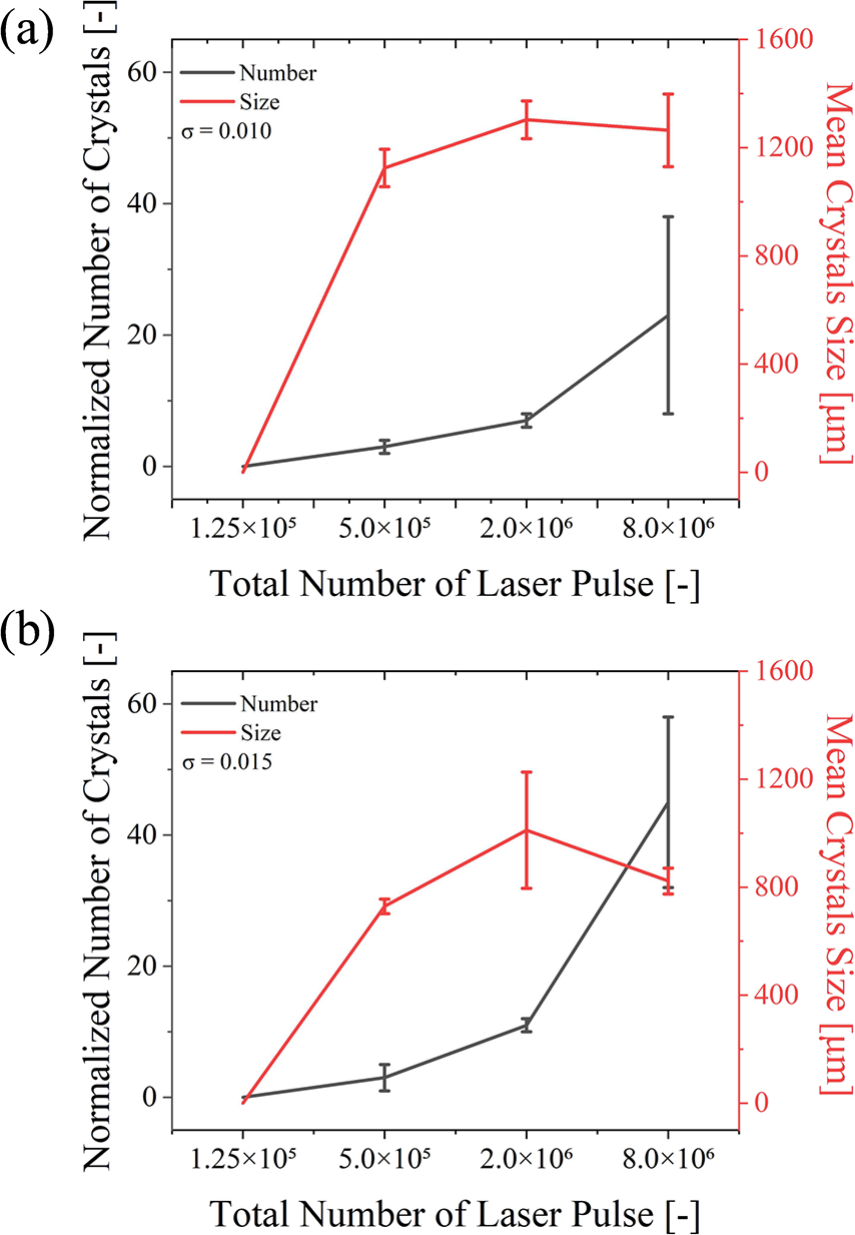
(a) Correlation graph of number and size of crystals vs total laser pulse number at supersaturations of 0.010. (b) Correlation graph of number and size of crystals vs total number of laser pulse numbers at supersaturations of 0.015. Error bars indicate standard deviations (*n* = 3).

The observation of a threshold number of pulses required for nucleation suggests that the mechanism involves cumulative interactions, where successive laser pulses contribute incrementally to reaching the activation energy for nucleation. Alternatively, the system may retain memory of the laser pulses through transient changes in the local environment, such as temperature gradients or cavitation effects, which persist and accumulate over time to facilitate nucleation. This behavior highlights the dynamic nature of laser-induced nucleation and warrants further investigation into the interplay between the laser energy and the local environment.

### 3.3 Laser Pulse Energy

The impact on number and size of crystals were investigated under laser pulse energy by 0.4, 1.0, and 2.1 μJ/pulse. The experiment was done for three samples at each condition. As shown in Figure 4, it was observed that for both concentrations, the number of crystals increased with the number of laser pulses, while the crystal size gradually decreased. For the solution with σ = 0.010, the average number increased from 2 to 74, while the mean size decreased from 3032.0 to 342.0 μm. For the solution with σ = 0.015, the average number increased from 2 to 124, and the mean size decreased from 3255.0 to 213.0 μm. The trend of the laser power effect on crystal number and size is relatively clear. However, it could be observed that the crystallization induced at 2.1 μJ/pulse is excessively small and broken, which was more like polycrystal (Figure S2 in the Supporting Information).

**Figure 4:**
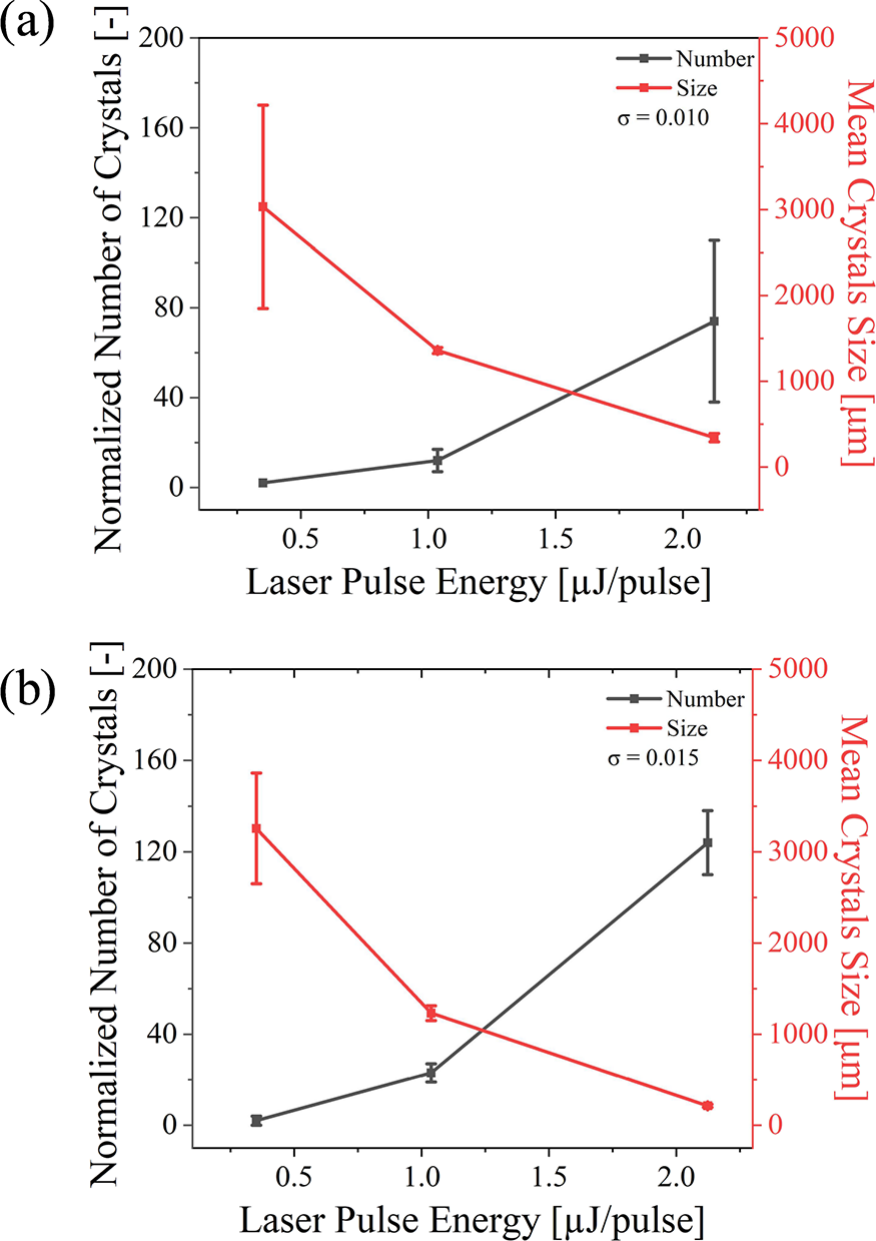
(a) Correlation graph of the number and size of crystals vs laser power at supersaturations of 0.010. (b) Plot of number and size of crystals vs laser power at supersaturations of 0.015. Error bars indicate standard deviations (*n* = 3).

The current commonly accepted view is that cavitation bubbles form during the femtosecond laser ablation in the supersaturated solution, which subsequently temporarily increases the local concentration of the solute, leading to a transient state.^29^ This highly concentrated region gradually dissipates, as NaClO_3_ molecules diffuse spontaneously. As a result, under lower pulse energy condition, crystal nuclei formed during this brief supersaturation period around smaller cavitation bubbles grow more slowly under lower supersaturation, which promotes the development of high-quality crystals. Laser pulses with high pulse energy not only form larger cavitation bubbles, increasing the nucleation frequency, but also affect a larger area, which may break the crystals induced by the previous laser pulse irradiation. Thus, we concluded that laser pulses with high pulse energy are necessary to obtain crystals of uniform size, but the laser pulses with too high pulse energy induce polycrystals.

### 3.4 Flow Rate

In a microfluidic flow system, the flow rate would impact the residence time, resulting in impacting the crystal size,^28^ and sometimes even crystal forms.^25^ Thus, the effect of the flow rate on femtosecond laser-induced crystal formation was also studied (as shown in Figure 5). The formation and behavior of cavitation bubbles are also influenced by the presence of solution flow in the microfluidic system.^36^ Under continuous flow conditions, convective transport affects the pressure distribution and energy dissipation around the cavitation region, potentially modifying bubble expansion and collapse dynamics. At higher flow rates, bubbles may be elongated or displaced downstream before full collapse, altering their interaction with the surrounding solute environment. While the primary factor governing nucleation remains the laser-induced energy deposition, flow conditions contribute to the overall crystallization dynamics by regulating solute availability and pressure variations in the microchannel. In the experiments investigating the effect of the flow rate on crystal number and size, the total irradiated solution volume was kept constant at 500 μL for all flow rate conditions. To achieve this, the flow time was adjusted inversely with the flow rate, ensuring that the same volume of solution was exposed to laser irradiation under each condition. This approach isolates the effect of the flow rate on crystal nucleation and growth dynamics while eliminating confounding factors related to differences in solution volume. For samples of both concentrations, under the same laser induction conditions, with the flow rate increasing, the number of crystals gradually increased, while the crystal size gradually decreased. The observed trend of increasing crystal number and decreasing crystal size with higher flow rates can be primarily attributed to the enhanced solute transport in the microfluidic system. At higher flow rates, solute replenishment near the laser-induced nucleation sites is more efficient, sustaining a higher local supersaturation and promoting nucleation. The resulting increase in nucleation events leads to more crystals competing for solute, ultimately reducing the final crystal size. Although shear flow in microfluidic channels can sometimes induce secondary nucleation, the flow conditions in our system do not generate sufficiently high shear forces to cause significant crystal fragmentation. Therefore, the dominant mechanism influencing crystal formation in our study is the control of solute availability and nucleation site density rather than shear-induced secondary nucleation As the flow rate increased from 100 to 2000 μL/min, for the solution with σ = 0.010, the average number increased from 3 to 41, and the mean size decreased from 9887.0 to 535.0 μm. For the solution with σ = 0.015, the average number increased from 1 to 106, and the mean size decreased from 4446.0 to 206.0 μm.

**Figure 5:**
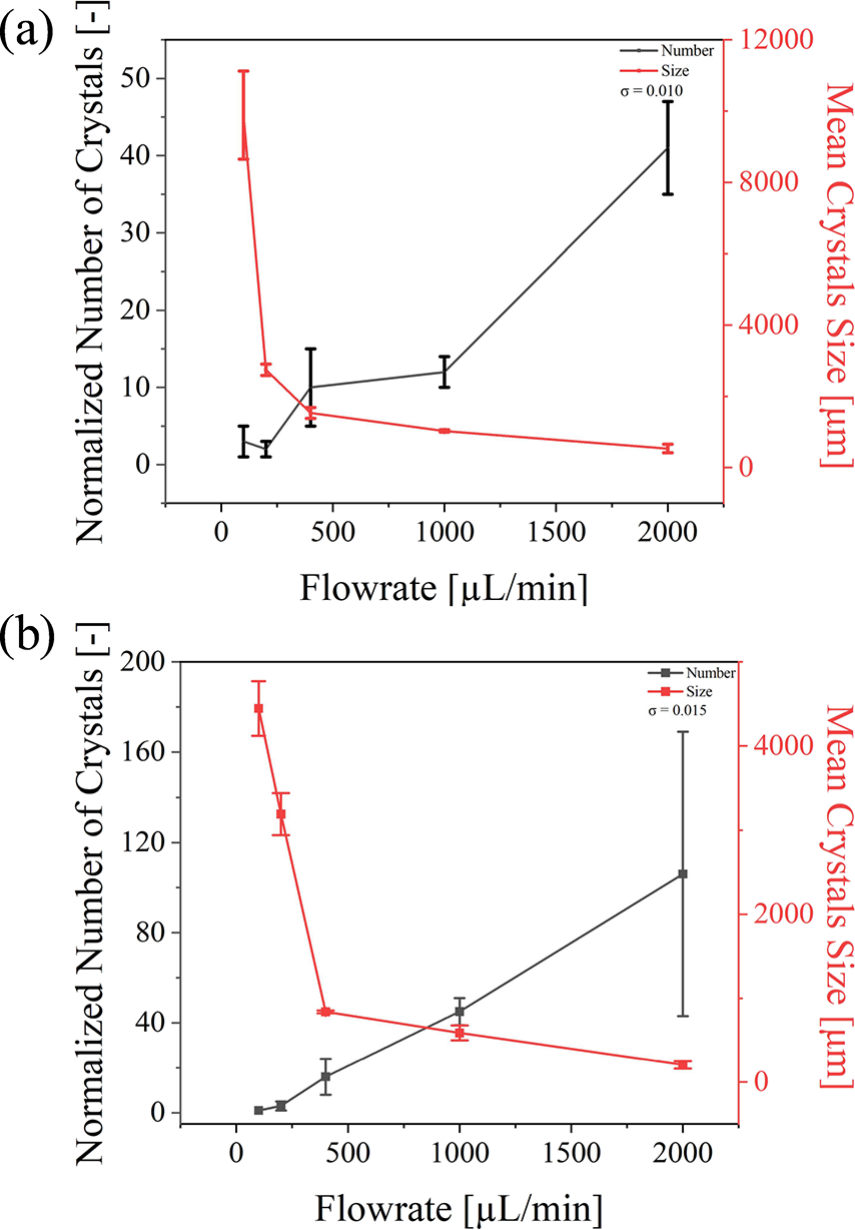
(a) Correlation graph of number and size of crystals vs flow rate at supersaturations of 0.010. (b) Correlation graph of number and size of crystals vs flow rate at supersaturations of 0.015. Error bars indicate standard deviations (*n* = 3).

The results indicate that the fluid velocity significantly affects the number and size of the crystals formed. Similar findings have been reported in other studies.^28,37^ When the flow rate is too low, typically less than 100 μL/min, the crystals tend to become overly dense, which can easily block the microfluidic channels. The reason why higher flow rates lead to a greater number of crystals may be that the continuous flow prevents localized solute depletion around the growing nuclei. At lower flow rates, stagnant fluid zones can create concentration gradients, which may hinder nucleation and crystal growth in the surrounding areas. Instead, they are quickly carried away by the fluid, allowing the nuclei to grow into crystals during the 24 h period. Additionally, femtosecond lasers can irradiate more solution at higher flow rates; more crystal nuclei are generated, resulting in a phenomenon where the number of crystals is proportional to the flow rate. When comparing our findings with the recent study by Ndukwe-Ajala et al.,^23^ which also investigated salt solutions using microfluidics and focused nanosecond laser, we observed several similarities. Their results demonstrated that the irradiated volume size is directly proportional to the nucleation rate. Furthermore, single-phase flow facilitates consistent crystal formation by maintaining a steady solute supply and preventing localized constraints caused by stagnant fluid zones. Although the microfluidic channel imposes physical limitations on the volume available for crystal growth, the continuous flow ensures uniform conditions for nucleation and growth. These conclusions align closely with our own observations. Thus, we concluded that increasing the flow rate is effective in inducing uniform crystallization.

## 4 Conclusions

This study, for the first time, enhanced the efficiency of femtosecond laser-induced crystallization in supersaturated solutions by using a microfluidic system, achieving a threshold crystallization concentration as low as σ = 0.002. By systematically varying the laser pulse energy, total pulse number, flow rate, and supersaturation, we established the correlation between crystal number and size, enabling sequential and tunable crystal generation in the microfluidic chip in the closed system. The system allows precise control over crystal sizes from approximately 200–3000 μm and numbers ranging from a single crystal to around one hundred. Compared to conventional static systems, the continuous flow environment of the microfluidic platform enhances mass and heat transfer and mitigates localized solute depletion and thermal effects. These improvements result in a scalable, dynamic process with high reproducibility, as demonstrated in Figures 2–5 and Table 1. Overall, our approach provides a significant advancement in femtosecond laser-induced crystallization, addressing key challenges such as finite volume, low throughput, and mass/heat transfer constraints while offering superior control over crystallization outcomes.

The observed inverse relationship between the number of crystals and the crystal size is attributed to solute competition and mass balance effects. As the nucleation density increases, a greater number of growing crystals deplete the available solute, limiting their individual growth. This effect is well established in crystallization studies, where high nucleation rates often lead to reduced final crystal sizes due to constrained solute availability and competition between growth and nucleation dynamics. Higher nucleation rates result in a greater number of nuclei competing for the available solute, thereby limiting individual crystal growth and leading to smaller crystal sizes. This theoretical framework aligns with the trends observed in Figures 2–5, where conditions promoting higher nucleation rates, such as increased laser pulse energy or flow rates, consistently produced smaller crystals. Incorporating this understanding emphasizes the importance of controlling nucleation rates to achieve the desired crystal sizes in laser-induced crystallization systems.

In addition to the effect of laser pulse energy on crystal habit, we observed that supersaturation, flow rate, and laser pulse number also significantly influenced the crystal morphology. At higher supersaturation levels, rapid nucleation resulted in polycrystalline structures, whereas lower supersaturation conditions favored the growth of well-defined single crystals. Similarly, the flow rate played a role in shaping the crystal habit, with higher flow rates promoting directional growth and elongated morphologies due to enhanced solute transport, while lower flow rates supported more uniform growth. Additionally, an increased number of laser pulses led to multiple nucleation events, often forming polycrystalline aggregates. These findings demonstrate that the combination of microfluidics and femtosecond laser-induced crystallization provides a tunable platform for controlling the crystal habit by adjusting key experimental parameters.

In our current setup, only a small fraction of the total flowing solution is directly irradiated by a femtosecond laser (∼0.1% of the total volume). This localized irradiation ensures precise nucleation control but results in a significant portion of the solution passing through without a direct laser interaction. Compared with nanosecond laser microfluidic crystallization, where larger beam profiles or different irradiation strategies may utilize a greater fraction of the flowing solution, this approach may be considered less efficient in terms of solute usage. Future improvements, such as optimizing the beam path, increasing the irradiated volume fraction, or implementing multiple irradiation zones within the microfluidic system, could enhance the overall efficiency of laser-induced nucleation while maintaining precise control over crystallization dynamics.

Microfluidics holds significant potential for advancing the efficiency of femtosecond laser-induced crystallization by enabling the laser trapping to be continuous on the supersaturation solution without heat accumulation. these different technique combinations. This is of great significance for the studies involving sensitive or costly solutes and solvents where a deeper understanding of crystallization is crucially desired.

## Supplementary Material



## Notes

Schematic diagram of the microfluidic device; comparison of crystallization outcomes, image of crystals, and photograph of the experimental setup (PDF)
