# Supplementary material for: Microfluidic Femtosecond Laser-Induced Nucleation of Supersaturated Aqueous Sodium Chlorate Solutions
Source: ACS Omega. 2025 Jun 23;10(27):28857–65. doi: 10.1021/acsomega.4c11633 (PMC12268445; doi:10.1021/acsomega.4c11633)
Supplement: Supplementary file 1 [file ao4c11633_si_001.pdf]

Supporting Information

Microfluidic Femtosecond Laser-induced Nucleation of Supersaturated Aqueous Sodium Chlorate Solutions

Liye Yang[1,2], Yoichiroh Hosokawa[1], Ming Li[3], Yuka Tsuri[1 *], Shaokoon Cheng[2*], Yaxiaer Yalikun [1*]

1. *Division of Materials Science, Graduate School of Science and Technology, Nara Institute of Science and Technology, Ikoma 630-0192, Japan;*
2. *School of Engineering, Macquarie University, Sydney, 2109, NSW, Australia;*
3. *School of Mechanical and Manufacturing Engineering, University of New South Wales, Sydney, NSW 2052, Australia*

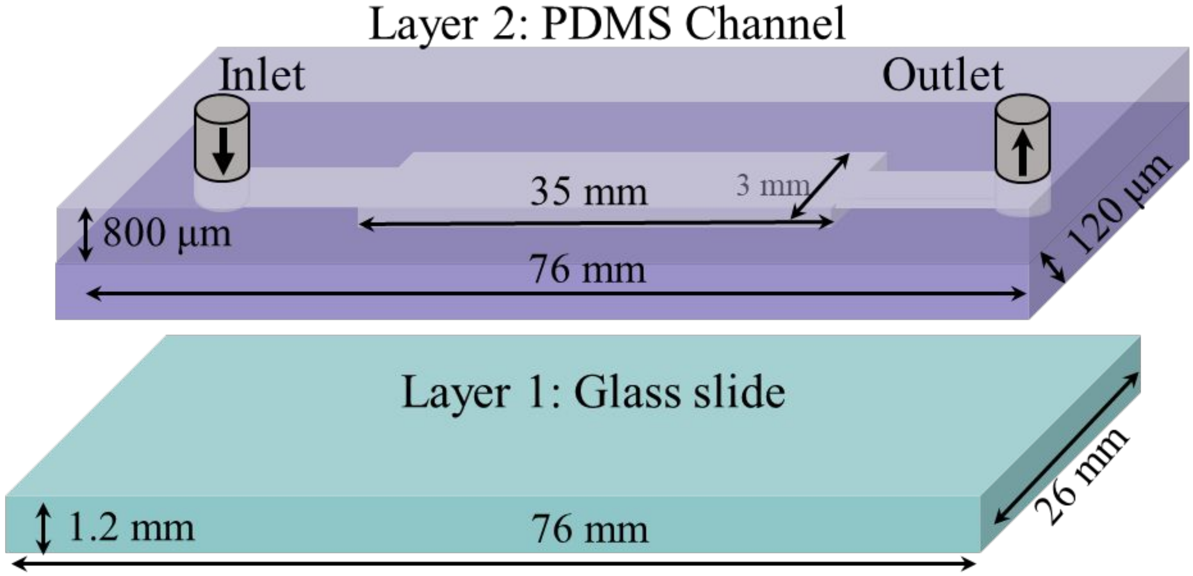

Figure S1: Schematic diagram of the microfluidic device used in this study. The device consists of two layers: (1) a glass slide (Layer 1) serving as the base, with dimensions of 76 mm × 26 mm, and (2) a polydimethylsiloxane (PDMS) layer (Layer 2) containing the microchannel structure. The microchannel dimensions are as follows: height = 0.12 mm, total length = 7.5 mm, and widths of various segments as labeled. The PDMS layer has a total thickness of 0.8 mm.

Table S1: Comparison of crystallization outcomes under different experimental conditions at supersaturation levels (σ=0.010 and σ=0.015). The results demonstrate that the combination of microfluidics and femtosecond laser-induced crystallization significantly improves crystallization efficiency, with higher success rates, controlled crystal sizes, and improved reproducibility compared to using microfluidics or femtosecond laser alone. The combined system achieves a 50% success rate at σ=0.010 and 100% success at σ=0.015, highlighting its superior performance in inducing and controlling crystallization.

<table>
<tr><th></th><th>Experimental Condition</th><th colspan="4">Crystallization Observed</th><th>Crystallization probability (%)</th><th>Crystal Size (Mean, μm)</th></tr>
<tr><td rowspan="3">σ = 0.010</td><td>Microfluidic chip flow only (200 μL/min)</td><td colspan="4">-</td><td>0%</td><td>N/A</td></tr>
<tr><td>Laser irradiation only (1.0 μJ/pulse) (No microfluidic chip flow)</td><td colspan="4">-</td><td>0%</td><td>N/A</td></tr>
<tr><td>Microfluidic chip flow and Laser irradiation (200 μL/min, 1.0 μJ/pulse, total pulse number 2.0×10^6)</td><td>-</td><td>+</td><td>-</td><td>+</td><td>50%</td><td>1360</td></tr>
<tr><td rowspan="3">σ = 0.015</td><td>Microfluidic chip flow only (200 μL/min)</td><td colspan="4">-</td><td>0%</td><td>N/A</td></tr>
<tr><td>Laser irradiation only (1.0 μJ/pulse) (No microfluidic chip flow)</td><td colspan="4">-</td><td>0%</td><td>N/A</td></tr>
<tr><td>Microfluidic chip flow and Laser irradiation (200 μL/min, 1.0 μJ/pulse, total pulse number 2.0×10^6)</td><td>+</td><td>+</td><td>+</td><td>+</td><td>100%</td><td>1231</td></tr>
</table>

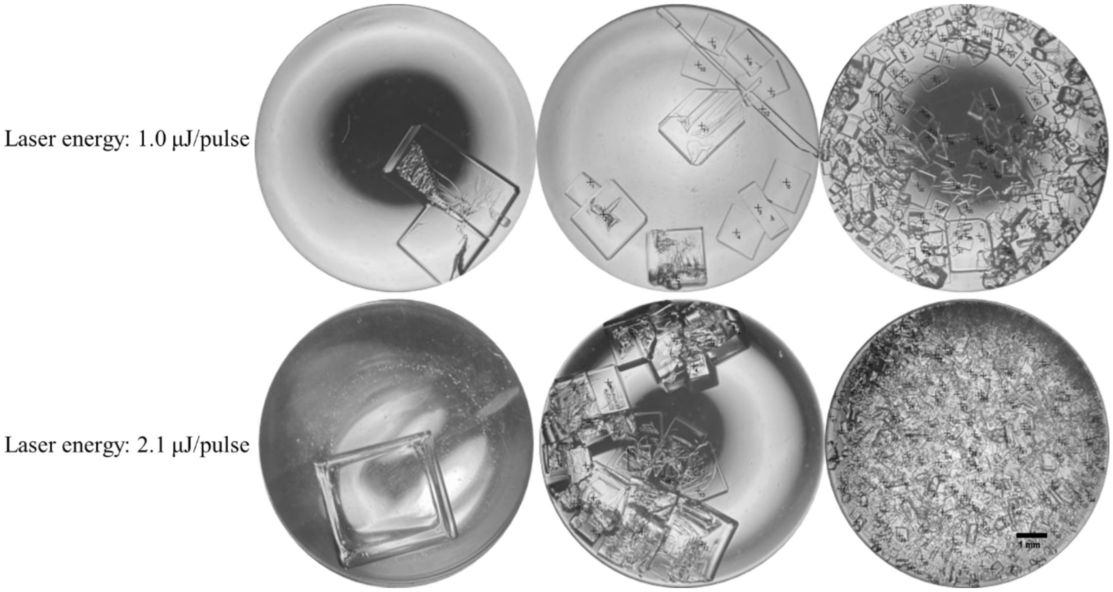

Figure S2: Representative image of crystals induced at 1.0 and 2.1 μJ/pulse in a solution with σ = 0.015, showing excessively small and broken morphology resembling polycrystalline structures. The image was captured using an optical microscope, and the crystal number and size were analyzed using ImageJ software.

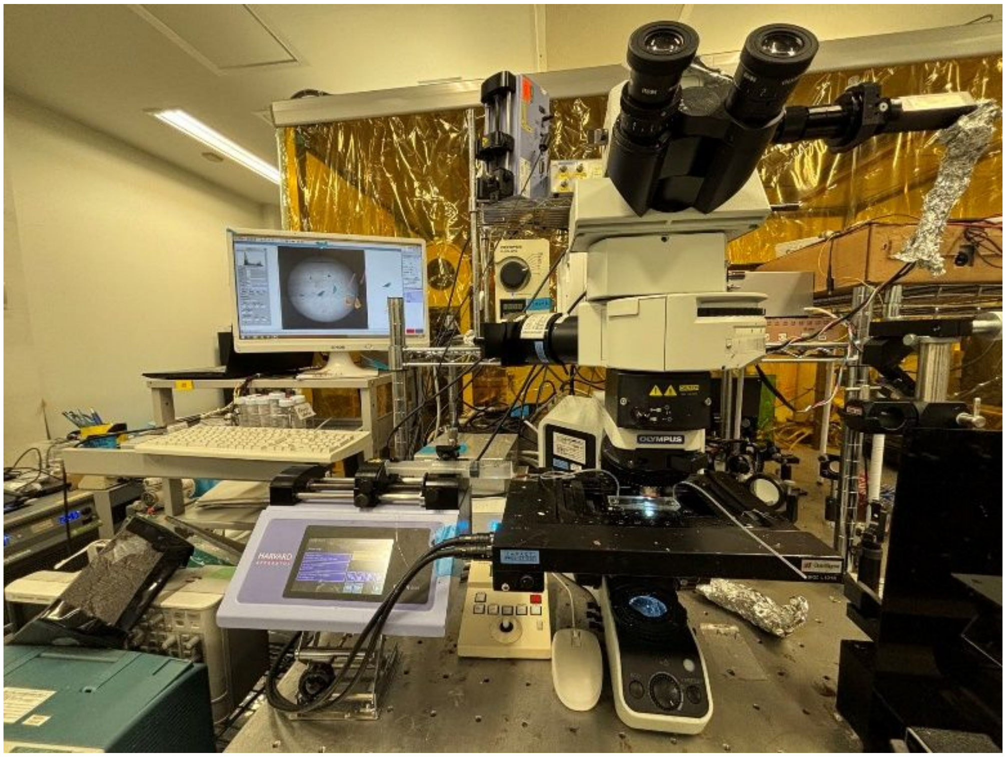

Figure S3: Photograph of the experimental setup used for femtosecond laser-induced supersaturated aqueous sodium chlorate solution crystallization in a microfluidic system.
